# The influence of a color themed HMI on trust and take-over performance in automated vehicles

**DOI:** 10.3389/fpsyg.2023.1128285

**Published:** 2023-07-13

**Authors:** Aboubakr el Jouhri, Ashraf el Sharkawy, Hakan Paksoy, Omar Youssif, Xiaolin He, Soyeon Kim, Riender Happee

**Affiliations:** ^1^Department of Cognitive Robotics, Faculty of Mechanical, Maritime and Materials Engineering, Delft University of Technology, Delft, Netherlands; ^2^Department of Human Centered Design, Faculty of Industrial Design Engineering, Delft University of Technology, Delft, Netherlands

**Keywords:** take-over request, trust, automated driving, HMI, driving simulator

## Abstract

**Introduction:**

SAE Level 3 is known as conditional driving automation. As long as certain conditions are met, there is no need to supervise the technology and the driver can engage in non-driving related tasks (NDRTs). However, a human driver must be present and alert to take over when the automation is facing its system limits. When such an emergency takes place, the automation uses the human machine interface (HMI) to send a take-over request (TOR) to the driver.

**Methods:**

We investigated the influence of a color themed HMI on the trust and take-over performance in automated vehicles. Using a driving simulator, we tested 45 participants divided in three groups with a baseline auditory HMI and two advanced color themed HMIs consisting of a display and ambient lighting with the colors red and blue. Trust in automation was assessed using questionnaires while take-over performance was assessed through response time and success rate.

**Results:**

Compared to the baseline HMI, the color themed HMI is more trustworthy, and participants understood their driving tasks better. Results show that the color themed HMI is perceived as more pleasant compared to the baseline HMI and leads to shorter reaction times. Red ambient lighting is seen as more urging than blue, but HMI color did not significantly affect the general HMI perception and TOR performance.

**Discussion:**

Further research can explore the use of color and other modalities to express varying urgency levels and validate findings in complex on road driving conditions.

## 1. Introduction

Development of Automated Vehicles (AV) has been the main focus of car manufacturers as well as tech companies. It is envisioned that AV could replace human driving, which could reduce traffic accidents and thus increase road safety. The implementation of AV could also benefit the “driver” by increasing comfort and enabling use of the driving time for work or leisure (Kyriakidis et al., 2015). The adoption of AV in day-to-day life is not only dependent on the performance of the automation. The ultimate goal for AV is to be broadly adopted in society. For this to happen, AV have to be trusted and accepted by the public.

The main focus of this study will be on SAE Level 3 automation, which is defined as “The sustained and operational design domain (ODD) specific performance by an automated driving system (ADS) of the entire dynamic driving task (DDT) with the expectation that the DDT fallback-ready user is receptive to ADS-issued requests to intervene, as well as to DDT performance-relevant system failures in other vehicle systems, and will respond appropriately” (SAE International, 2021). This means that when the automation is activated, the driver can take the eyes off the road and perform non-driving related tasks (NDRTs). However, this requires high levels of trust defined as “the attitude that an agent will help achieve an individual's goals in a situation characterized by uncertainty and vulnerability” (Lee and See, 2004). When the automation reaches its limits, the automation requests the driver to take over control of the vehicle. This request initiated by the vehicle is called a take-over request (TOR; Lu et al., 2016). The TOR is conveyed by a Human Machine Interface (HMI) which relays information from a machine or system to a person (Stouffer et al., 2011).

With the increasing advancements and availability of automated vehicles, the number of studies related to HMI in automated vehicles has been rising. A review by Ayoub et al. (2019) showed that take-over, acceptance and trust, interaction with other road users, and design methodology were studied extensively in the last decade. Several studies demonstrated effects of TOR HMI modality and information on performance (Politis et al., 2015; Petermeijer et al., 2017; Zhang et al., 2019; Naujoks et al., 2021). Simple sounds and lights and ambient lighting can alert the driver and are often sufficient to elicit safe take-overs. Zhang et al. (2019) and Kim et al. (2021) found that TOR presented only through the visual modality yielded the longest reaction time, whereas adding the auditory modality reduced the reaction time. A range of studies on various HMI elements demonstrated that advanced HMI informing users of the automation operation, and desired driver actions through images, written text or spoken text, outperform basic HMI in terms of trust as well as performance (Kim et al., 2021). Trust was enhanced adding speech based maneuver information (Forster et al., 2017) and vibrotactile spatial information related to traffic objects (Sonoda and Wada, 2017). Trust in automation and take-over performance were enhanced showing the TOR on a display (Löcken et al., 2020) and situation awareness was increased through a LED bar indicating the direction of conflicts and the vehicle trajectory (Yang et al., 2018). Ambient lighting provides information without demanding much cognitive workload and does not distract users' from their primary tasks (Matthews et al., 2004). It has a positive influence on, amongst others, perceived safety and interior functionality (Caberletti et al., 2010). Mirnig et al. (2023) showed that ambient light interfaces can improve mode awareness and reduce unsafe NDRT engagement in Level 3 automation. Several studies showed effects of various design aspect of ambient lighting in automated vehicles such as ambient lighting position (Feierle et al., 2020) or patterns of ambient lighting (Borojeni et al., 2016). Some literature addresses symbol color (Campbell et al., 2018) and color contrast with background (Naujoks et al., 2019). However, to the best of our knowledge, the effect of the ambient lighting color has not been studied for HMI in automated driving.

In general, a red color, which has a large wavelength, stimulates physical performance (Hill and Barton, 2005). Red can convey the urgency and safety critical aspect of the TOR, while colors as blue would suggest a lower urgency (Friedrich and Vollrath, 2022). A higher urgency can promote a rapid TOR response (Politis et al., 2015) but may also induce high stress levels hampering trust, acceptance and take-over performance. This indicates that red would be beneficial for the reaction time. On the other hand, red undermines cognitive performance and blue is perceived as more calming and trustworthy (Elliot et al., 2007). This indicates that blue colors, which have a smaller wavelength, let the driver be more comfortable and relaxed, which would lead to a more calm and safe take-over.

As outlined above, ample studies report effects of HMI on take-over reaction time but few report the effect on perceived safety and trust in automation. In most cases the color of the HMI display and ambient lighting is assigned rather than researched. Hence this paper investigates the following main hypotheses:

- A color themed informative HMI with ambient lighting will increase the trust in AV (Hypothesis 1a) and improve the take-over reaction time and quality (Hypothesis 1b).
- A red themed HMI will result in a higher urgency and therefore a shorter reaction time as compared to a blue-themed HMI (Hypothesis 2).

In order to validate these hypotheses, a color themed HMI including ambient lighting and an informative display was designed. The influence of the presence and color of the HMI was evaluated considering trust and take-over performance in a driving simulator experiment.

## 2. Methods

A color themed HMI was designed including a display at the midconsole, ambient lighting and sound whereas a basic HMI, which only uses sound, was used as baseline. The choices that led to the design of the HMI and the experiment are given below.

### 2.1. Participants

Forty-five participants were recruited via various channels to take part in this experiment, of which 14 were female and 31 were male. All had at least 2 years of driving experience and were between 18 and 28 years old. The participants were evenly divided in three test groups based on Gender, Driving Experience and results from the pre-trust in automation questionnaire. The group that was tested with a basic HMI that is not color themed was called “Baseline”. The group that was tested with the red themed HMI was called “Red”. The last group that was tested with a blue themed HMI was called “Blue”.

### 2.2. Apparatus

**Figure 1:**
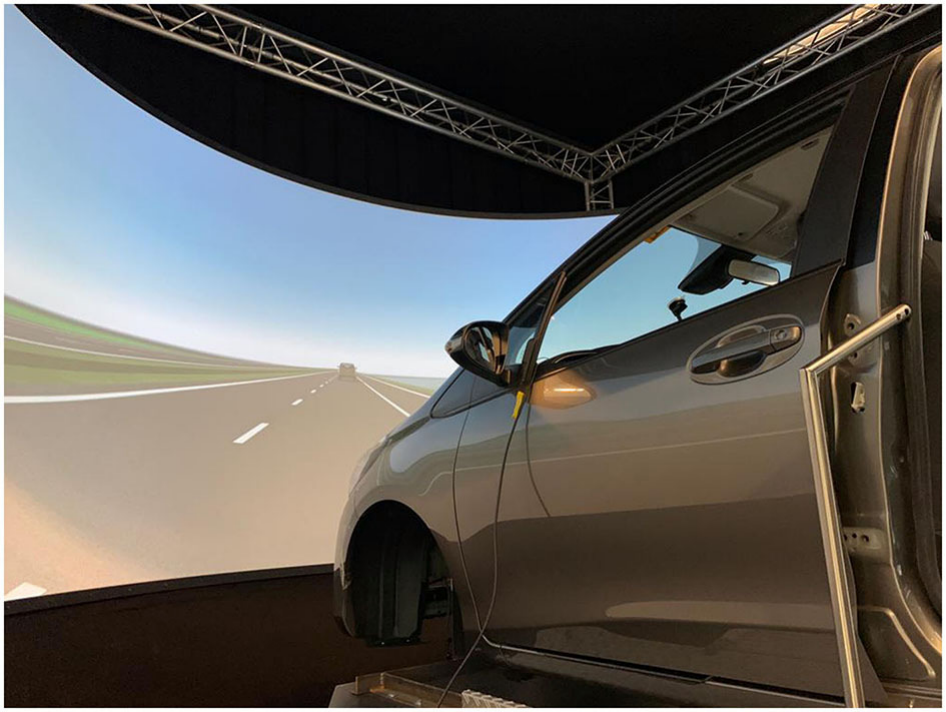
DAVSi driving simulator.

**Figure 2:**
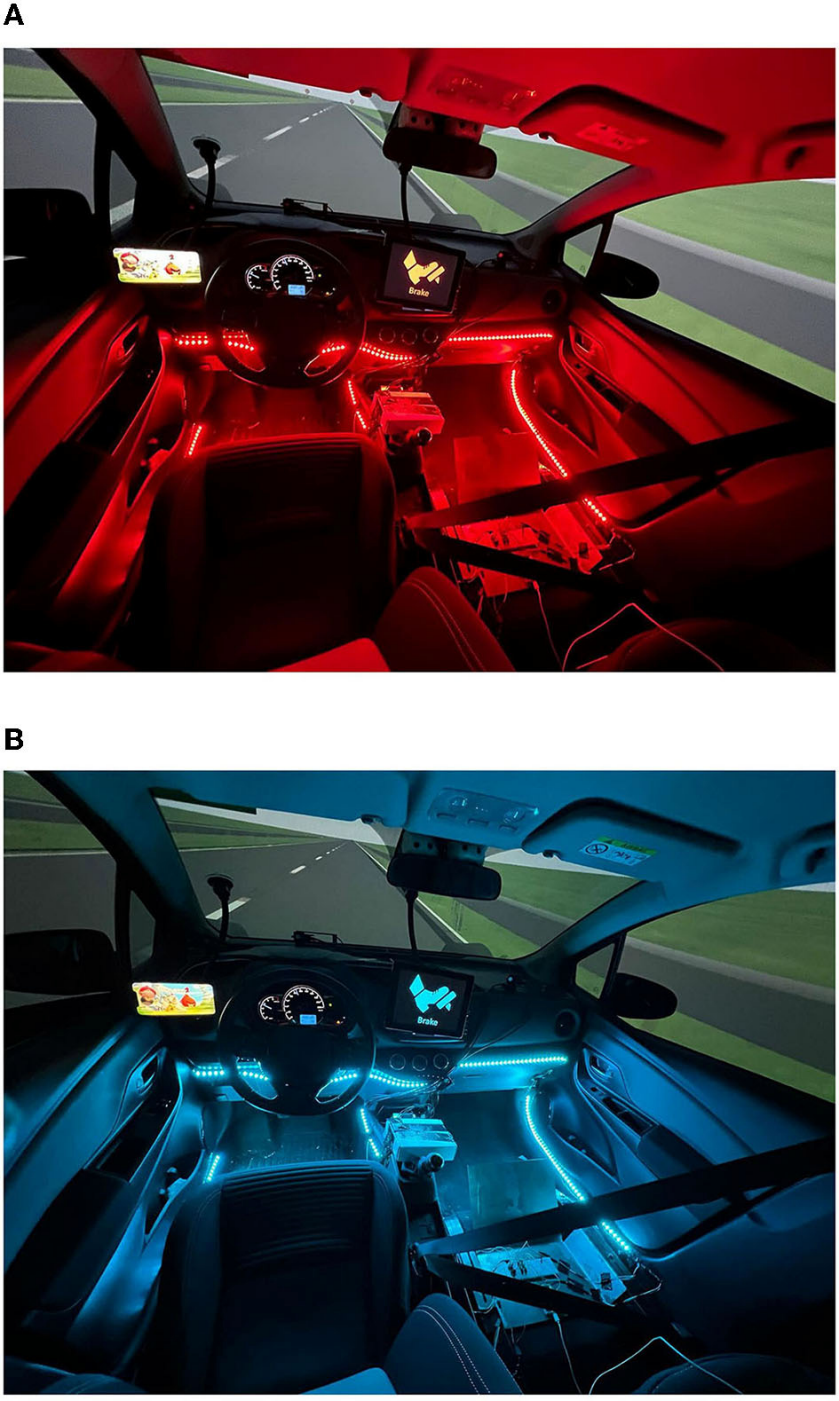
Two color-themed HMIs with ambient lighting, HMI display at the midconsole, and NDRT left of the steer. **(A)** Red themed HMI. **(B)** Blue themed HMI.

The experiment took place at the intelligent vehicles group at the Delft University of Technology, and was conducted using the Delft Advanced Vehicle Simulator (DAVSi) (Figure 1; Khusro et al., 2020). The mock-up vehicle placed in the simulator, a 2013 Toyota Yaris, includes a driver and passenger seat. The instrument cluster is functioning and shows the speed and the RPM of the vehicle. The projection screen that shows the driving environment is placed in front of the mock-up vehicle and shows a 200° field of view. IPG CarMaker 8.0.1 was used to generate the environment and traffic of the scenario. IPG movie was used to display the graphics on the screen. The color themed HMIs used a 10.9-inch display in the center console and ambient lighting to show the TOR request (Figures 2A, B). A smartphone on the left side of the steering wheel was used for the NDRT (Figures 2A, B). The ambient lighting consisted of multiple led strips controlled by an Arduino receiving commands from DAVSi via a series port. The IPG driver model was used to realize automated driving. During take-over drivers were expected to control speed by braking.

### 2.3. HMIs

Three different HMIs were designed and used in the experiment. The HMI of the baseline group consisted of only an auditory interface, in which no ambient lighting or display was shown during a TOR. The HMI of the color themed groups consisted of the same auditory and a visual interface as shown in Table 1 and Figures 2A, B.

**Table 1:** Overview of the different HMIs.

**HMI**	**Baseline**	**Red**	**Blue**
Auditory	Generic sounds	Generic sounds	Generic sounds
Lighting	None	Red ambient lighting	Blue ambient lighting
Display	None	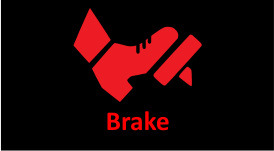	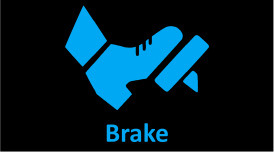

#### 2.3.1. Auditory interface

There were two transition-related sounds in the vehicle (see Supplementary material). When the AV issued a TOR, a short sound of 1.5 s was repeated until the driver intervened. This sound is called “TOR-sound.” As the driver was engaging in an NDRT and was visually distracted, the TOR sound was expected to be the first TOR sign that would be noticed by the participant. When the AV resumed automated driving after the TOR, a short sound was played for 1.5 s. This sound is called “automation-start-sound.” After hearing this sound the driver was expected to continue with the NDRT as the automation took back control. The two sounds were designed for informing AV activation and requesting take-over. Alarm levels, usefulness, and appropriateness with situations of the two sounds were validated in our previous study (Kim et al., 2022). Results have shown that the two sounds were appropriately designed for their driving context.

#### 2.3.2. Visual interface

##### 2.3.2.1. Color

The three different HMIs could be distinguished by the presence and color of a visual HMI and ambient lighting. The basic HMI used sound only. The other two HMIs, used the colors red and blue in both the HMI display and as ambient lighting.

##### 2.3.2.2. Display

For the color themed HMIs, the visual TOR was shown on the mid-console display which was placed just below the window. A drawing of a brake pedal together with the text “Brake” indicated that the participant had to take over control by braking.

##### 2.3.2.3. Lighting

For the color themed HMIs, ambient lighting was added on several points in the interior, below eyesight, so that the driver could notice the lights without getting distracted or blinded. The red themed HMI contained red ambient lighting and the blue themed HMI contained blue ambient lighting (Figures 2A, B).

Both the display and the ambient lighting were activated at the start of each TOR and remained active until the automation took back control. When seeing the lighting, the driver was expected to check the HMI display and take over control by braking.

### 2.4. Scenario

**Figure 3:**
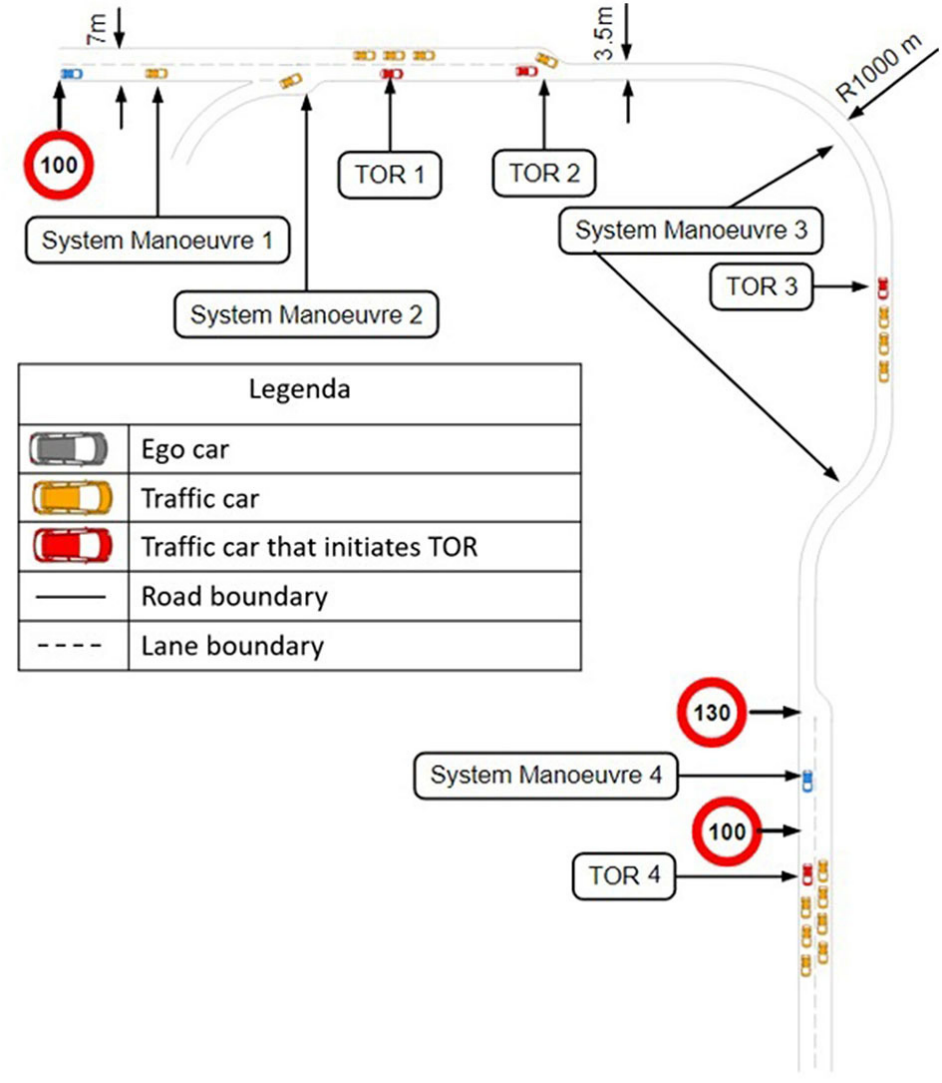
Road map of the scenario.

The driving scenario had eight separate situations which took place on a highway. Four situations were handled by the automation, whereas four others included a TOR. The TOR were designed to create a new experience in each TOR requiring an original action rather than triggering a preprogrammed response. The drive was initiated with SAE Level 3 automation, starting from standstill and accelerating to the speed limit which is 100 km/h in most parts and 130 km/h in others (Figure 3). The 8 situations can be subdivided into two categories, system maneuvers and TORs. The system maneuvers were handled by the automation. The TORs were to be handled by the participant, where in all TOR braking was the appropriate intervention. However, when participants did not brake in time, the automation intervened if the time to collision (TTC) became < 2 s, and prevented collisions. To ensure that the participants would not be confused by the situations, a distinction between system maneuvers and TORs has been made. Take-over situations only included braking (speed control) while system maneuvers included steering. During the whole scenario, there was foggy weather with a visibility of 300 m. This created a somewhat more critical situation to test trust in automation. However, the 300 m always enabled timely visual detection of other vehicles well before the TOR.

The first phase of the scenario was designed to make the participants acquainted with the simulator, the automation and the TOR. This phase consisted of two system maneuvers. Following this phase, the first and second TOR took place. After these TORs, the third system maneuver, the third TOR, the fourth system maneuver, and the fourth TOR took place, respectively (Figure 3). In the next sections, the system maneuvers and TORs will be explained in detail. To minimize the influence of one situation on the other, there was a minimum of 3 min in between every traffic situation. Whenever, the AV was in automated state, the participant had to engage in an NDRT, which was playing Angry bird, a visual-motor task without sound, on a smart phone mounted left of the steering wheel (Figure 2). The NDRT was self-paced and interruptible, so participants could pause it whenever necessary to check the surrounding (Naujoks et al., 2017).

#### 2.4.1. System maneuvers

There were three types of system maneuvers.

**Figure 4:**
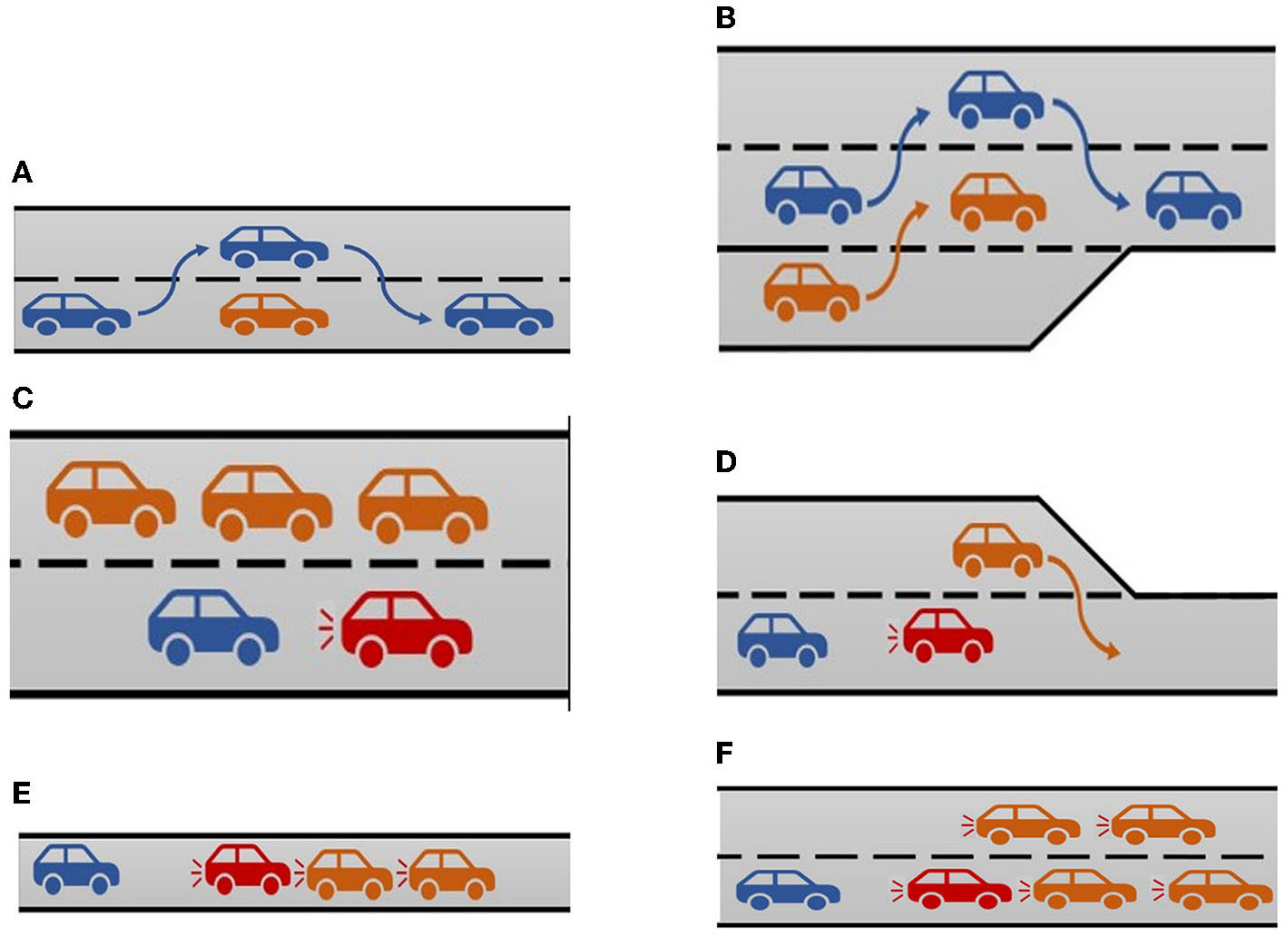
System maneuvers and take-over requests. **(A)** System maneuver 1 and 4 [ego car (blue) is overtaking a slower moving traffic car (orange)]. **(B)** System maneuver 2 [ego car (blue) is changing lanes to create space for a merging traffic car (orange)]. **(C)** TOR 1 [Traffic car (red) brakes hard, ego car (blue) cannot change to the left lane due to the traffic cars (orange), so a TOR is initiated. The participant is expected to brake.]. **(D)** TOR 2 [Traffic car (orange) cuts off the red traffic car, the traffic car (red) brakes which leads to a TOR for the driver which is represented by the ego car (blue)]. **(E)** TOR 3 [Traffic jam on a one-lane road caused by stationary traffic cars (orange), traffic car (red) brakes which results in a TOR for the ego car (blue)]. **(F)** TOR 4 [Traffic jam on a two-lane road caused by stationary traffic cars (traffic), traffic car (red) brakes which results in a TOR for the ego car (blue)].

- The ego vehicle overtakes a vehicle driving slower on the right lane in System maneuver 1 and 4 (Figure 4A).
- The ego vehicle overtakes a vehicle suddenly merging in front from the insertion lane in System maneuver 2 (Figure 4B).
- Steering of the ego vehicle to make it follow the curvature of the road in System Manouvre 3.

#### 2.4.2. TORs

In total there were four TORs where a sudden change of events caused the AV to request a TOR.

- The first TOR (TOR 1 in Figure 3) took place on a two-lane road. The ego car had a constant speed of 100 km/h for 2 km before the automation suddenly noticed that the traffic car in front of the ego car was braking. The distance to the traffic car was around 100 m and even though there was a left lane, an overtake was no option because of multiple cars passing on the left lane with a speed of 110 km/h (Figure 4C). Therefore, the ego-car driver had to take over control and achieve hard braking. After braking, the traffic car in front of the ego car had a speed of 40 km/h which resulted in the ego car eventually overtaking the traffic car.
- The second TOR (TOR 2 in Figure 3) took place on a piece of road where two lanes merged into one (Figure 4D). The ego car was driving on the right lane and there was a traffic car driving in front of it. As the end of the left lane approached, a traffic car on the left lane tried to overtake the traffic car in front of the ego car (Figure 4D). The traffic car was forced to brake suddenly to a speed of 50 km/h and the automation requested a TOR. The distance to the traffic car in front was around 80 m, and an overtake was no option because of the merging left lane. The ego-car driver had to achieve hard braking to prevent an accident. After the TOR, the traffic car accelerated to a speed of 110 km/h.
- The third TOR (TOR 3 in Figure 3) took place on a one-lane road. The ego car encountered a traffic jam (Figure 4E). The distance to the traffic car in front was around 150 m, and an overtake was not possible as there was no left lane. The only safe outcome was achieved by hard braking. After the TOR, the traffic cars accelerated to a speed of 115 km/h, leaving the ego car behind.
- The fourth and last TOR (TOR 4 in Figure 3) took place on a two-lane road. Just like the previous TOR, the ego car encountered a traffic jam (Figure 4F). The distance to the traffic car in front was around 150 m. An overtake was not possible as there was a traffic jam on the left lane too. The ego-car driver had to achieve hard-braking. After the TOR, the traffic cars accelerated to a speed of 115 km/h, leaving the ego car behind.

### 2.5. Procedure

Each participant had to read an instruction with information about the steps that would take place before, during and after the simulator drive. After this instruction, the participants were asked to fill in a pre-drive questionnaire, which contained an explanation about SAE Level 3 automation (Table 2). The pre-drive questionnaire was used to test the familiarity and trust of the participant regarding SAE Level 3 automation before taking part in the research, and was used to create balanced participant groups for the 3 HMIs. Filling in the pre-drive questionnaire took about 10 min. After finishing the pre-drive questionnaire the participants took place in the simulator. The participants went through the designed scenario which took around 30 min to finish. During the simulator drive, two questions were asked after each TOR. After finishing the drive, participants were asked to fill in a post-drive questionnaire which took about 15 min to finish (Table 3). The post-drive questionnaire was used to measure the trust in the automation after having experienced the automation. The whole process took around 50–60 min per participant.

**Table 2:** Pre-drive questionnaire divided in three groups.

**Group**	**Question**
G1—Automation experience	Have you ever experienced Adaptive Cruise Control?
	Have you ever experienced Lane-Assist?
	Have you ever experienced a combination of Adaptive Cruise Control and Lane-assist?
	Have you ever experienced Tesla's Auto-pilot?
G2—Trust in automation	How safe would you feel in an SAE Level 3 automated vehicle?
	Are SAE Level 3 automated vehicles dangerous? (Reverse coded)
	To what extent would you trust an SAE Level 3 automated vehicle?
	Would you feel fine handing over control to an SAE Level 3 Automated Vehicle?
G3—NDRT	To what extent are you willing to do Non-Driving Related Tasks, such as using your phone, while using the driving automation system?

Participants could rate every question from 1 (strongly disagree) to 5 (strongly agree). This questionnaire was loosely based on Nordhoff et al. (2021).

**Table 3:** Post-drive questionnaire divided in groups with their corresponding references.

**Group**	**Questions**	**Reference source**	**Original questions**
G4—Experience with automation	The system's performance matches my expectations of an automated vehicle	Modified after Forster et al. (2017)	The system's performance matches my expectations of a CAD system
G5—Trust in automation	I trust the system to safely operate in the next drive.	Forster et al. (2017)	I trust the system to safely operate in the next drive
		I am convinced of the system.	Forster et al. (2017)	I am convinced of the system
		The system is safe.	Modified after Waytz et al. (2014)	The system provides safety
		The system is dangerous.	Forster et al. (2017)	The system is dangerous
		The system is trustworthy.	Forster et al. (2017)	The system is trustworthy.
		The system is working accurately.	Modified after Forster et al. (2017)	I suppose the system works accurately
G6—Trust process	I am familiar with the system.	Jian et al. (2000)	I am familiar with the system.
		I trust the system to warn me in time before a take-over situation.	New item	
		The system is deceptive.	Jian et al. (2000)	The system is deceptive.
		I trust the system during system maneuvers.	Modified after Forster et al. (2017)	I trust the system's mode of operation during system maneuvers
G7—Trust purpose	I feel fine handing over control to the system.	Forster et al. (2017)	I feel fine handing over control to the system
		The system is a reliable partner.	Forster et al. (2017) (modified by Jian et al., 2000)	The system is a reliable partner
		The vehicle seems to be intelligent.	Forster et al. (2017)	The vehicle seems to be intelligent.
G8—HMI driving task	I would prefer more communication from the system.	Modified after Nordhoff et al. (2023)	The information conveyed by the interface is unambiguous & The information conveyed by the interface is clear to me
		My driving tasks during a take-over were clear.	Modified after Forster et al. (2017)	Driving task responsibility was explicitly clarified
		The information provided by the user interface is sufficient.	Modified after Nordhoff et al. (2023)	The information conveyed by the interface is clear to me
		The user interface clearly conveyed if the automation was working or not.	Modified after Nordhoff et al. (2023)	The information conveyed by the interface is recognizable
G9—HMI feeling	The user interface is calming.	Modified after Verberne et al. (2012)	To what extent would using this ACC system evoke the feeling calmness?
		The user interface is comprehensible.	Modified after Nordhoff et al. (2018)	The user interface would not take long to learn
		The user interface is complex.	Modified after Nordhoff et al. (2018)	The user interface would not take long to learn
		The user interface is predictable.	Modified after Nordhoff et al. (2018)	Is easy to understand
G10—Ambient color urgency Perception	How did the color of the ambient lighting make you feel? [scale from 1 (calming) to 5 (urging)]	New item	–

Participants could rate every question from 1 (strongly disagree) to 5 (strongly agree).

### 2.6. Measurements

Objective measurements of driver performance were gained during the simulator drive while subjective measurements were obtained through pre-drive and post-drive questionnaires, and questions asked during the drive after each TOR.

#### 2.6.1. Objective measurement

The objective data can be divided into two groups, the take-over time and take-over quality. The take-over time was measured by the reaction time, between the initiation of the TOR and the first brake or gas input from the participant (Gold et al., 2013). The take-over quality was measured by the percentage of successful TORs. A TOR was considered successful if the automation did not intervene by braking (automation intervened if the TTC became < 2 s).

#### 2.6.2. Subjective measurement

The pre-drive questionnaire (Table 2) was used to assess participants' earlier experiences with AV (G1), their initial thoughts on trust and acceptance in AV (G2) and willingness to perform NDRT in AV (G3) and was based on earlier studies (e.g., Nordhoff et al., 2021, 2023) where they proved to be effective to assess automation experience.

During the simulator drive, the participant were asked two questions after each TOR:

*G11—How stressful was this TOR on a scale from 1 to 5?*

*G12—How urgent was this TOR on a scale from 1 to 5?*

The data gathered from these questions were used to assess the take-over perception of the participants. The questions were asked directly after each TOR so the participants had a clear memory of their perception.

The post-drive questionnaire filled in after the simulator drive has been used to obtain the participants' thoughts on the automation as experienced. Questions were derived from a wide range of literature sources (e.g., Nordhoff et al., 2021, 2023) and complemented with two new items as detailed in Table 3. The advantage of having pre- and post-drive questionnaires is that apart from drawing conclusions on the absolute trust in automation, conclusions about the increase in trust can be drawn.

## 3. Results

**Figure 5:**
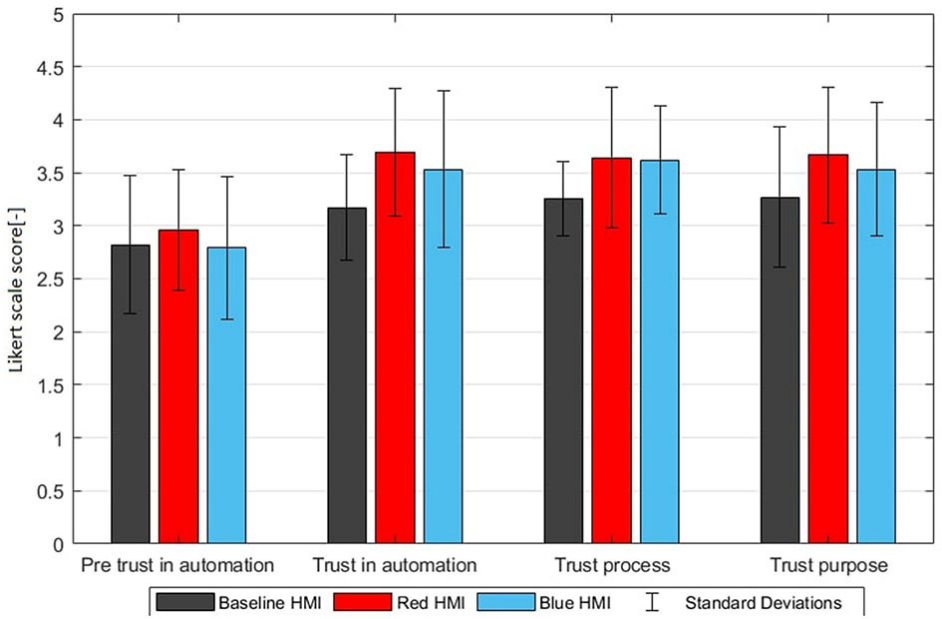
Trust increase visualized by the pre-drive trust in automation G2, post-drive trust G5, trust process G6, and trust purpose G7 (mean ratings with standard deviations on a Likert scale from 1 to 5).

**Figure 6:**
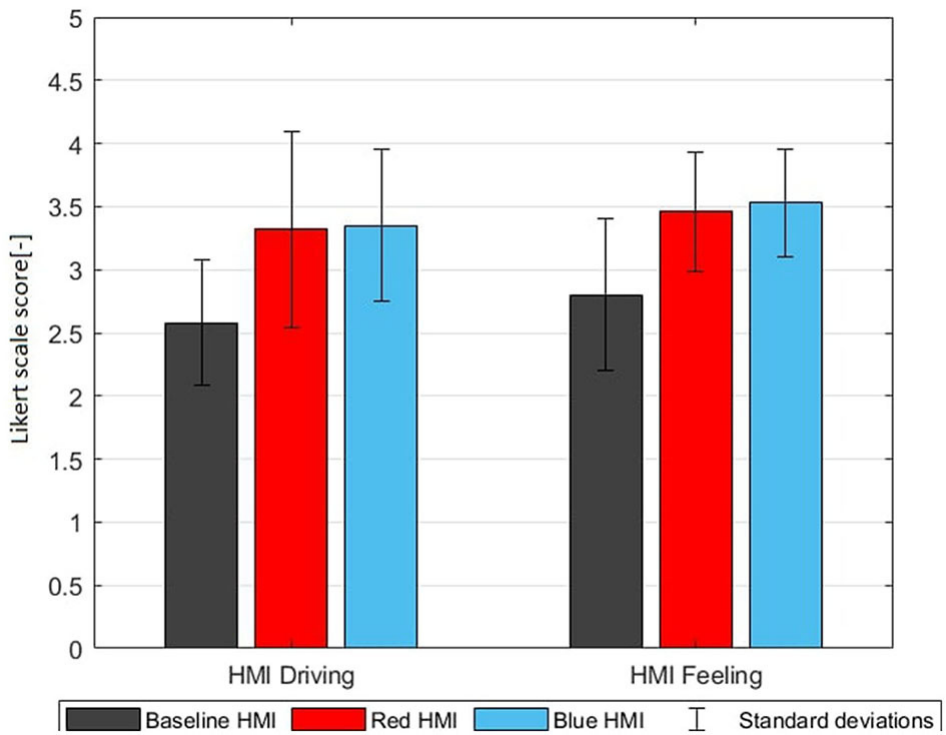
HMI driving task G8 and HMI feeling G9 (mean ratings with standard deviations on a Likert scale from 1 to 5).

**Figure 7:**
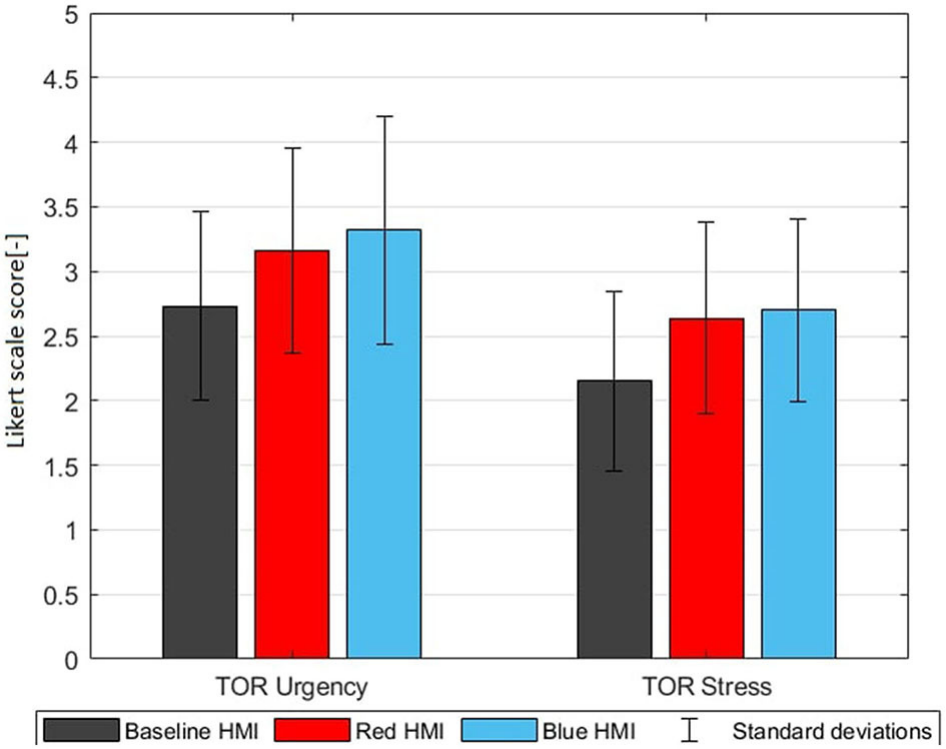
TOR perception of urgency G11 and stress G12 (mean ratings with standard deviations on a Likert scale from 1 to 5).

**Figure 8:**
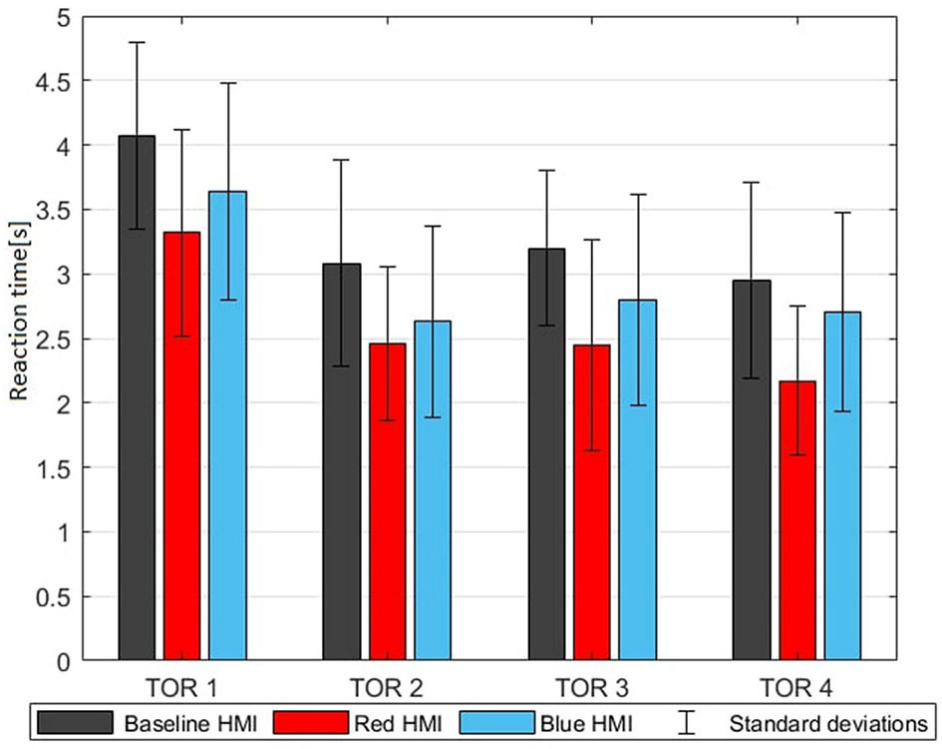
TOR reaction times G17 for every TOR (means with standard deviations).

All participants completed the full experiment, responded adequately to most TORs, and did not intervene in the automated system maneuvers. Results with the three HMIs are presented in Tables 4–6 and Figures 5–8.

**Table 4:** Summary of the pre-drive and post-drive questionnaire data.

**Question group**	**HMI**	**M**	**SD**	***p*-values**
G2—Pre-trust in automation	Baseline	2.82	0.65	*p*_*BC*_ = 0.837
	Red	2.96	0.57	*p*_*RB*_ = 0.460
	Blue	2.79	0.67	G3—Pre-NDRT	Baseline	2.53	1.19	*p*_*BC*_ = 0.561
	Red	3.00	1.36	*p*_*RB*_ = 0.316
	Blue	2.53	1.13	G4—Experience with automation	Baseline	3.20	0.68	*p*_*BC*_ = 0.003
	Red	4.00	0.96	*p*_*RB*_ = 1.00
	Blue	3.93	0.96	G5—Trust in automation	Baseline	3.17	0.50	*p*_*BC*_ = 0.016
	Red	3.69	0.60	*p*_*RB*_ = 0.529
	Blue	3.53	0.74	(G5-G2)—Trust increase	Baseline	0.35	0.57	*p*_*BC*_ = 0.016
	Red	0.73	1.25	*p*_*RB*_ = 0.529
	Blue	0.74	1.05	G6—Trust process	Baseline	3.25	0.35	*p*_*BC*_ = 0.007
	Red	3.64	0.66	*p*_*RB*_ = 0.905
	Blue	3.62	0.51	G7—Trust purpose	Baseline	3.27	0.66	*p*_*BC*_ = 0.113
	Red	3.67	0.64	*p*_*RB*_ = 0.569
	Blue	3.53	0.63	G8—HMI driving task	Baseline	2.58	0.50	*p*_*BC*_ < 0.001
	Red	3.32	0.78	*p*_*RB*_ = 0.911
	Blue	3.35	0.60	G9—HMI Feeling	Baseline	2.80	0.60	*p*_*BC*_ < 0.001
	Red	3.46	0.47	*p*_*RB*_ = 0.677
	Blue	3.53	0.43	G10—Ambient color urgency perception	Red	3.92	0.95	*p*_*RB*_ = 0.001
	Blue	2.07	0.80	

Means and standard deviations were calculated for every HMI along with the corresponding *p*-values.

**Table 5:** Summary of the questionnaire responses on the take-over perception asked after each TOR.

**Question group**	**HMI**	**M**	**SD**	***p*-values**
G11—TOR urgency	Baseline	2.73	0.73	*p*_*BC*_ = 0.042
	Red	3.16	0.79	*p*_*RB*_ = 0.613
	Blue	3.32	0.88	G12—TOR Stress	Baseline	2.15	0.70	*p*_*BC*_ = 0.036
	Red	2.64	0.74	*p*_*RB*_ = 0.829
	Blue	2.70	0.71	

Means and standard deviations were calculated for every HMI along with the corresponding *p*-values.

**Table 6:** Percentage of successful TOR results for every HMI.

	**Baseline**	**Red**	**Blue**	***p*-values**
G18—Percentage	7.5	26	11.25	*p*_*BC*_ = 0.060
Successful TORs				*p*_*RB*_ = 0.892

Results for question groups are presented as an average over items in each question group (see Tables 2, 3). Before averaging, items representing negative perceptions were reverse coded (“How dangerous are SAE Level 3 automated vehicles,” “The system is deceptive,” “The user interface is complex”).

Results show clear positive effects of the presence of the color themed visual HMIs with few effects of HMI color. The significance of these effects was evaluated using two *p*-values. The *p*_*BC*_ value, calculated with a two-sample *t*-test, compares the baseline group with the two color themed HMIs combined, to evaluate the difference between the simple auditory and the more advanced HMIs. The *p*_*RB*_ value, also calculated with a two-sample *t*-test, compares the red group with the blue group, to assess the effect of HMI color.

Questions G2 and G3 from the pre-drive questionnaire were used to create balanced participant groups for the three HMIs. This was successful as differences between groups for G2 and G3 were insignificant (see Table 4).

### 3.1. Trust and HMI experience

Table 4 shows pre-drive and post-drive questionnaire responses for the three HMIs. The post-drive questionnaire shows substantial benefits of the color themed HMIs for G4-G9. These benefits are significant for all question groups except for G7. However in G4–G9 no significant differences are found between the blue themed and red themed HMI. The only significant effect of HMI color is the ambient color perception (G10) where, as expected, red was perceived as much more urging (3.92) compared to blue (2.07) on a scale from 1 to 5. However, this higher urgency associated with ambient color did not have negative or positive effects on the overall perception of trust and HMI (G4–G9). This demonstrates that both color themed HMIs significantly enhanced the automation experience and trust and that these HMIs were positively evaluated. However, these subjective benefits of the color themed HMIs did not differ between the red and blue themed HMIs.

### 3.2. Take-over perception

After each TOR, every participant answered questions about their perception of the TOR. Since the urgency and stress per TOR is not relevant for this paper, the results of the four TORs averaged per HMI group are reported in Table 5. The color themed HMIs induced a significantly higher urgency and stress (*p*_*BC*_ = 0.042). The *p*_*RB*_ values however are very high (>0.6) which indicates that differences between red and blue are insignificant. Thus, both color themed HMIs create a similar TOR urgency and stress in G11 and G12 even though the ambient color red was perceived as more urging in G10.

### 3.3. Take-over performance

Table 7 shows the TOR reaction time. TOR data of four participants were not saved. With the first and fourth TOR, two participants did not react and thus no reaction time was collected. The second and third TOR had three participants which did not react. This missing data was not taken into account in any calculations of reaction time. The color themed HMIs reduced the TOR reaction time in TOR1–TOR4 and this effect was significant for TOR1, TOR3, and the average over TOR1–TOR4. The red themed HMI reduced the reaction time with an average of 0.42 s against baseline whereas the blue themed HMI reduced the reaction time with only 0.15 s. However this difference between red and blue themed HMIs was not significant (*p*_*RB*_ = 0.156). The quality of the take-over is measured by the success rate. This is a percentage that shows how many take-overs were completed without any intervention of the automation. This success rate is shown in Table 6 and indicates that the automation intervened in a majority of TORs. Apparently the time-window offered to react was rather short while the perceived urgency to intervene was modest (the urgency was around 3 on a scale from 1 to 5: see G11 in Table 5). This only emerged analyzing all results. The majority of the participants thought they were handling the take-over themselves, while in reality it was the automation that intervened. This issue was most apparent with the baseline HMI, whereas the transition between manual and automated driving was more clear for the color themed HMIs since the ambient lighting turned off when the automation took over. The red themed HMI led to most successful TORs but effects of HMI on TOR quality were not significant.

**Table 7:** Summary of the reaction time (in seconds) for every HMI.

**TOR number**	**HMI**	**M [s]**	**SD [s]**	***p*-values**
G13—TOR 1reaction time	Baseline	4.07	0.72	*p*_*BC*_ = 0.046
	Red	3.32	0.80	*p*_*RB*_ = 0.364
	Blue	3.64	0.84	G14—TOR 2 reaction time	Baseline	3.08	0.80	*p*_*BC*_ = 0.063
	Red	2.46	0.60	*p*_*RB*_ = 0.532
	Blue	2.63	0.74	G15—TOR 3 reaction time	Baseline	3.20	0.60	*p*_*BC*_ = 0.015
	Red	2.45	0.82	*p*_*RB*_ = 0.304
	Blue	2.80	0.82	G16—TOR 4 reaction time	Baseline	2.95	0.76	*p*_*BC*_ = 0.063
	Red	2.17	0.58	*p*_*RB*_ = 0.060
	Blue	2.70	0.77	G17—Average	Baseline	3.23	0.47	*p*_*BC*_ = 0.023
TOR reaction time	Red	2.81	0.60	*p*_*RB*_ = 0.156
	Blue	3.08	0.77	

Means and standard deviations were calculated for every HMI along with the corresponding *p*-values.

## 4. Discussion

This study investigated the influence of a color themed HMI on trust and take-over performance in an automated vehicle. Forty-five participants were divided in three groups, each with a different HMI. Every participant had to perform four TORs in a 30 min drive, during which their performance was measured. Before, during and after the drive, participants filled in questionnaires to assess their increase in trust.

### 4.1. Trust and HMI experience

With the color themed HMIs the TORs were perceived as more urgent and more stressful as compared to the baseline HMI (Figure 7). The higher urgency can be seen as positive and the higher stress is not alarming with a mean around 2.7 on a scale from 1 to 5. However, the higher stress could be associated with the additional information to be processed with the advanced HMIs.

With all three HMIs trust in automation was higher after the experiment (Figure 5) and this trust increase was significantly higher with the color themed HMIs. While the trust of the baseline group only increased with 0.35, the trust of the red and blue themed HMI groups increased with 0.73 and 0.74, respectively. Furthermore, the color themed HMIs scored better on “Trust Process (G6),” “Trust purpose (G7),” “HMI Driving task (G8),” and “HMI Feeling (G9)” and this effect was significant for G6, G8, and G9.

These positive effects of the ambient lighting and the informative visual HMI on trust strongly support Hypothesis 1a. Lee and See (2004) defined trust as “the attitude that an agent will help achieve an individual's goals in a situation characterized by uncertainty and vulnerability.” The positive effects of HMIs can be jointly attributed to the ambient lighting attracting the attention when the TOR is issued and the HMI display instructing the driver to brake. Apparently the non-intrusive ambient lighting combined with the specific instructions on the display are well appreciated. These positive effects of the informative visual HMI and ambient lighting align with (Yang et al., 2018; Zhang et al., 2019; Löcken et al., 2020) who have shown how visual HMI using ambient lighting increase trust (Löcken et al., 2020) and support faster driver reactions (Yang et al., 2018; Zhang et al., 2019). Furthermore, our study advances the existing literature by demonstrating the combined effects of ambient lighting and informative visual HMIs, which was not addressed in previous studies such as Yang et al. (2018) and Löcken et al. (2020).

### 4.2. Take-over performance

Regarding the take-over performance (G13, G14, G15, G16, G17) in Figure 8, two results stand out. The reaction times in the first TOR are significantly higher in comparison to the following TORs with all three HMIs. This indicates that participants were learning how to react to the TORs, which is a common finding (Gold et al., 2018). The red themed HMI elicited the shortest reaction times in all four TORs. This reaction time benefit was significant comparing the color themed HMIs to baseline which supports Hypothesis 1b and can (similar to the effect on trust) be jointly attributed to the ambient lighting attracting the attention when the TOR is issued and the HMI display instructing the driver to brake. Our findings on the benefits of the red-themed HMI are consistent with previous research on color and urgency perception. Friedrich and Vollrath (2022) found that red leads to a significantly shorter reaction time than yellow in situations requiring quick reactions with unmanned aircraft systems so it was suggested to use red for safety critical indications. The shorter reaction time with the red-themed HMI is in line with Hypothesis 2 but the difference between red and blue was not significant. In line with Hypothesis 2, the red themed HMI also led to most successful TORs but effects of HMI on TOR quality were also not significant. This calls for the collection of more data including more participants and varying the actual urgency of the events. The automation performance can also be enhanced. The percentage of successful TORs (G18) indicates that the automation intervened in a majority of cases. This could have been alleviated through an even earlier TOR which however may not always be technically feasible. This could also be alleviated through a later but more aggressive intervention by the automation but this would presumably reduce perceived safety, trust, and motion comfort. In our study the automation intervened if the TTC became < 2 s. However this is overly conservative as TCC ignores accelerations. This could be resolved using the enhanced time to collision (ETTC) to control the intervention. ETTC takes in consideration the decelelration of both vehicles, which is relevant in our scenarios (Happee et al., 2017).


(1) $$\begin{array}{l} TTC=\frac{dx}{v} \end{array}$$

(2) $$ETTC=\{\begin{array}{cc} \frac{dx}{v}, & \text{if }a=0\\ \frac{v-\sqrt{v^{2}+2a\cdot dx}}{-a}, & \text{if }a\neq 0 \end{array}$$

### 4.3. Marginal effects of HMI color

The difference between the baseline and the color themed HMIs is substantial across a majority of results which demonstrates the ability of the selected experimental conditions and measures to assess the benefits of HMIs to enhance human performance and trust in automated driving. The differences between the red and blue themed HMIs, however, are small. The only significant difference between red and blue regards the ambient color perception (G10; Table 4). While red is perceived as urging, blue tends to be a more calming color (e.g., Elliot et al., 2007). Benefits of the red interface were insignificant in the current data but showed the expected trend (Hypothesis 2) with shorter reaction times (G13–G17) and more successful TORs (G18). This suggests that HMI color affects the perception of urgency rather than (or more strongly than) the actual TOR response.

### 4.4. Limitations and future work

We tested 3 groups of participants between 18 and 28 years old with at least 2 years of driving experience. Driving experience is a key factor in both trust and safe driving performance (Jin et al., 2020) and age affects risk perception and driving skill. The limited sample size may explain the insignificance of several effects of HMI color. Testing a larger and broader population sample is recommended to further establish benefits of color themed HMIs. Here also other colors and combinations of multimodal TOR HMIs can be explored including mixed reality (Riegler et al., 2020). The range of scenarios and urgency levels can be expanded, and users can develop their interpretation of HMI color over prolonged exposure. Effects of color could become significant testing more events but the current data shows limited effect sizes comparing red or blue HMIs. Potentially more promising would be the use of varying colors within HMIs using gradually changing colors with red signaling the highest urgency. TOR timing could be enhanced using ETTC rather than TTC as outlined in Section 4.2. Additionally, studying the effects of long exposure to different HMI colors could provide valuable insights into how users adapt and interpret HMI color over time. This would allow for a more comprehensive understanding of the impact of color and HMI design on trust and performance in automated driving scenarios. Finally, HMIs can be tested in actual vehicles on tracks and public roads and measures can be extended to further probe the understanding of HMI information and AV operation in realistic complex driving conditions.

## 5. Conclusions

This driving simulator study demonstrates significant benefits of a color themed HMI:

- The advanced color themed HMI with informative display and ambient lighting contributes more to the trust in automated vehicles in comparison to the baseline auditory HMI. Participants found the color themed HMI more trustworthy, they understood their driving tasks better and they found the HMI more pleasant to work with.
- The advanced color themed HMI outperforms the baseline HMI in case of TOR. Participants who experienced the color themed HMI found that the TORs were more urgent and stressful, which led to a reduced reaction time.
- The red ambient lighting is perceived as more urging while blue is perceived as calming, but effects of color on TOR performance and trust were not significant.

The obtained results could contribute to acceptance of automated vehicles. An advanced color themed HMI can improve trust in automated vehicles and contribute to safety in TOR.

## Data availability statement

The raw data supporting the conclusions of this article will be made available by the authors, without undue reservation.

## Ethics statement

The studies involving human participants were reviewed and approved by HREC Committee of Delft University of Technology. The patients/participants provided their written informed consent to participate in this study.

## Author contributions

AJ, AS, HP, and OY: conceptualization, data curation, formal analysis, methodology, validation, visualization, and writing—original draft. XH, SK, and RH: supervision, conceptualization, methodology, and writing—review and editing. All authors contributed to the article and approved the submitted version.

## Supplementary material

Two sounds representing the auditory cues and one video recording the whole experiment process.
